# Interaction of quorum signals with outer membrane lipids: insights into prokaryotic membrane vesicle formation

**DOI:** 10.1111/j.1365-2958.2008.06302.x

**Published:** 2008-06-09

**Authors:** Lauren Mashburn-Warren, Jörg Howe, Patrick Garidel, Walter Richter, Frank Steiniger, Manfred Roessle, Klaus Brandenburg, Marvin Whiteley

**Affiliations:** 1 The University of Texas at AustinAustin TX, 78712, USA; 2 Forschungszentrum Borstel, Leibniz-Zentrum für Medizin und BiowissenschaftenD-23845 Borstel, Germany; 3 Martin-Luther-Universität Halle-Wittenberg, Institut für Physikalische ChemieD- 06108 Halle, Germany; 4 Friedrich-Schiller-Universität Jena, Elektronenmikroskopisches Zentrum der Medizinischen FakultätD-07743 Jena, Germany; 5 European Molecular Biology Laboratory EMBLHamburg Outstation c/o DESY, 22603 Hamburg, Germany

## Abstract

Bacteria have evolved elaborate communication strategies to co-ordinate their group activities, a process termed quorum sensing (QS). *Pseudomonas aeruginosa* is an opportunistic pathogen that utilizes QS for diverse activities, including disease pathogenesis. *P. aeruginosa* has evolved a novel communication system in which the signal molecule 2-heptyl-3-hydroxy-4-quinolone (Pseudomonas Quinolone Signal, PQS) is trafficked between cells via membrane vesicles (MVs). Not only is PQS packaged into MVs, it is required for MV formation. Although MVs are involved in important biological processes aside from signalling, the molecular mechanism of MV formation is unknown. To provide insight into the molecular mechanism of MV formation, we examined the interaction of PQS with bacterial lipids. Here, we show that PQS interacts strongly with the acyl chains and 4′-phosphate of bacterial lipopolysaccharide (LPS). Using PQS derivatives, we demonstrate that the alkyl side-chain and third position hydroxyl of PQS are critical for these interactions. Finally, we show that PQS stimulated purified LPS to form liposome-like structures. These studies provide molecular insight into *P. aeruginosa* MV formation and demonstrate that quorum signals serve important non-signalling functions.

## Introduction

*Pseudomonas aeruginosa* is a ubiquitous Gram-negative bacterium and a frequent cause of opportunistic infections in immunocompromised individuals. *P. aeruginosa* is a primary colonizer of the lungs of individuals with the genetic disorder cystic fibrosis, where approximately 80% of individuals over 8 years of age are chronically infected. *P. aeruginosa* is also a common cause of nosocomial infections, including intensive care unit pneumonia, and these infections are often life-threatening as a result of the inherent antibiotic resistance of this bacterium. *P. aeruginosa* pathogenesis is complex and involves a wide array of extracellular factors critical for colonization and disease progression. Many of these factors are controlled by a cell density-dependent process termed quorum sensing (QS). QS involves production and sensing of small extracellular signalling molecules that allow a bacterium to monitor and respond to its own cell density. *P. aeruginosa* QS utilizes multiple signalling molecules, including 2-heptyl-3-hydroxy-4-quinolone (termed the Pseudomonas Quinolone Signal or PQS), which collectively allow the bacteria to communicate and co-ordinate their group activities (Pesci *et al*., 1999; Parsek and Greenberg, 2000). QS controls expression of approximately 500 genes in *P. aeruginosa* (Schuster *et al*., 2003; Wagner *et al*., 2003), including many that are required for virulence in multiple plant, mammalian and insect models (Jander *et al*., 2000; Pearson *et al*., 2000)., The QS paradigm involves secretion of signalling molecules into the extracellular milieu where they diffuse between cells to allow co-ordinated group behaviours. Unlike many other QS signalling molecules, PQS is extremely hydrophobic, making its diffusion into the extracellular milieu unlikely. Our laboratory recently demonstrated that PQS does not follow the classical QS signal tenet, but is instead trafficked within the *P. aeruginosa* population via membrane vesicles (MVs) (Mashburn and Whiteley, 2005). MVs are produced by most Gram-negative bacteria and arise through bulging and pinching off of the outer membrane. Similar to the outer membrane, MVs are bilayered with an outer leaflet of lipopolysaccharide (LPS) and an inner leaflet of phospholipids (Chatterjee and Das, 1967; Deich and Hoyer, 1982; Kahn *et al*., 1982; Pettit and Judd, 1992; Kadurugamuwa and Beveridge, 1995; Beveridge *et al*., 1997; Li *et al*., 1998; Beveridge, 1999; Fiocca *et al*., 1999). Aside from PQS, MVs serve as trafficking vehicles for a wide array of biologically relevant molecules, including bacterial toxins, DNA, antibiotic-resistance determinants and antimicrobial compounds (Kuehn and Kesty, 2005; Mashburn-Warren and Whiteley, 2006).

**Fig. 1:**
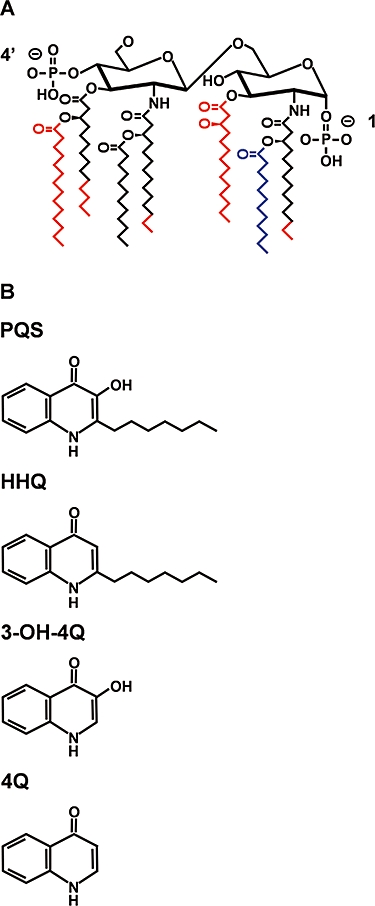
A. Structure of lipid A of *S. minnesota* R60 and *P. aeruginosa* PA14. Black represents shared structural components, substituents in red are specific to *S. minnesota* and substituents in blue are specific to *P. aeruginosa*. Unlike other *Salmonella* spp., R60 primarily possesses a free 4′-phosphate that is not modified with aminoarabinose (Boll *et al*., 1994; St Swierzko *et al*., 2000). Although not included, the 1-phosphate of R60 may be modified with phosphoethanolamine. B. Structures of quinolones used in this study: PQS, HHQ, 3OH-4Q and 4-quinolone (4Q).

Although the biological relevance of MVs has been noted for over 20 years, mechanistic insights into MV formation are limited. Our laboratory recently provided evidence that PQS is required for MV formation in *P. aeruginosa* (Mashburn and Whiteley, 2005), thus implicating a small signalling molecule as a key player in MV formation. Based on these observations, we proposed a model for *P. aeruginosa* MV formation in which PQS interacts with the LPS component of the outer membrane to initiate MV formation (Mashburn-Warren and Whiteley, 2006; 2008). LPS is composed of lipid A, core oligosaccharides and the polysaccharide O-antigen. The lipid A portion of LPS serves as the lipid anchor and is commonly composed of fatty acids, sugars and phosphate groups (Fig. 1A). Adjacent lipid A molecules in the outer membrane exhibit charge repulsion as a result of close proximity of the terminal phosphates and are stabilized *in vivo* by divalent cation salt bridges (Beveridge, 1999).

The LPS structures for many bacteria are known; however, few have been utilized experimentally to examine interactions with small molecules. LPS from *Salmonella minnesota* strain R60 has been used extensively to examine interactions of small molecules with the bacterial outer membrane. *S. minnesota* R60 possesses lipid A with a chemical structure similar to *P. aeruginosa*, although R60 LPS does not contain O-antigen (Fig. 1A). To examine the roles of PQS in MV formation and test our model that PQS interacts with lipid A, biophysical techniques were used to examine the interactions of PQS with LPS from *P. aeruginosa* and *S. minnesota* R60. Our results demonstrate that PQS interacts more strongly with LPS than the phospholipid component of the outer membrane. Specifically, PQS interacts with the 4′-phosphate and acyl chains of lipid A. We also provide evidence that the third position hydroxyl of PQS and the length of the alkyl side-chain are critical for mediating these interactions.

## Results

### PQS alters the acyl chain melting transition of LPS

**Fig. 2:**
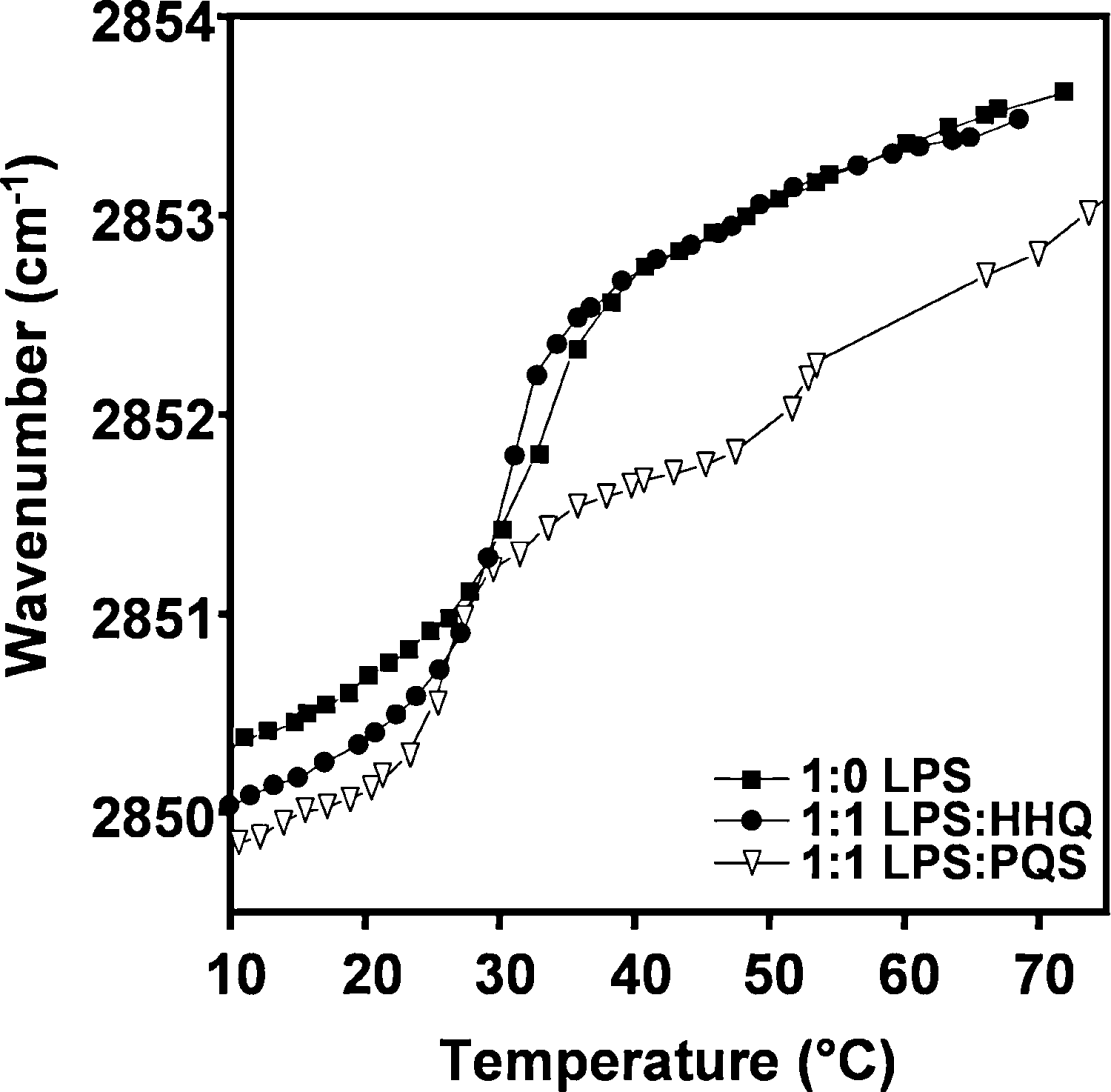
Phase transition of LPS is affected by PQS and not HHQ. Gel-to-liquid crystalline phase transition of the acyl chains of *S. minnesota* R60 LPS was measured by infrared spectroscopy alone, with an equimolar ratio of PQS and an equimolar amount of HHQ. Temperature scans were performed between 10°C and 70°C with a heating rate of 0.6°C min^−1^. Plotted is the peak position of the asymmetric stretching vibrational band of the methylene groups versus temperature, which is a sensitive measure of the acyl chain order. Data represent duplicate measurements.

The gel to liquid crystalline phase transition from a well-ordered (gel) into an unordered (liquid crystalline) state is a characteristic property of all membrane lipids that occurs as the temperature increases. For well-studied enterobacterial lipid A, such as that from *S. minnesota* R60, this phase transition lies in the range of 30–37°C, depending on the length of the carbohydrate moiety (Seydel *et al*., 1993a). As all molecules vibrate at a characteristic frequency, infrared (IR) spectroscopy can be utilized to study the phase transition of lipid A by examination of this vibrational spectrum. The peak position of the asymmetric stretching vibration of well-ordered lipid A occurs at 2850 cm^−1^ and increases as the gel-like state transitions to the liquid crystalline state. To investigate their effects on the acyl chains of lipid A, PQS and the non-hydroxylated PQS derivative 2-heptyl-4-quinolone (HHQ, Fig. 1B) were added to *S. minnesota* R60 LPS and examined by IR spectroscopy. The results indicate that HHQ alters the phase transition of LPS slightly, but at 37°C not at all (Fig. 2). In contrast, PQS interacted strongly with LPS by decreasing the acyl chain fluidity (strong decrease of the wave numbers), thus inhibiting the transition of LPS from a well-ordered (gel) into an unordered (liquid crystalline) state. This inhibition was most prominent at temperatures above 30°C, indicating that PQS influences the acyl chains of lipid A by making them more ordered.

**Fig. 3:**
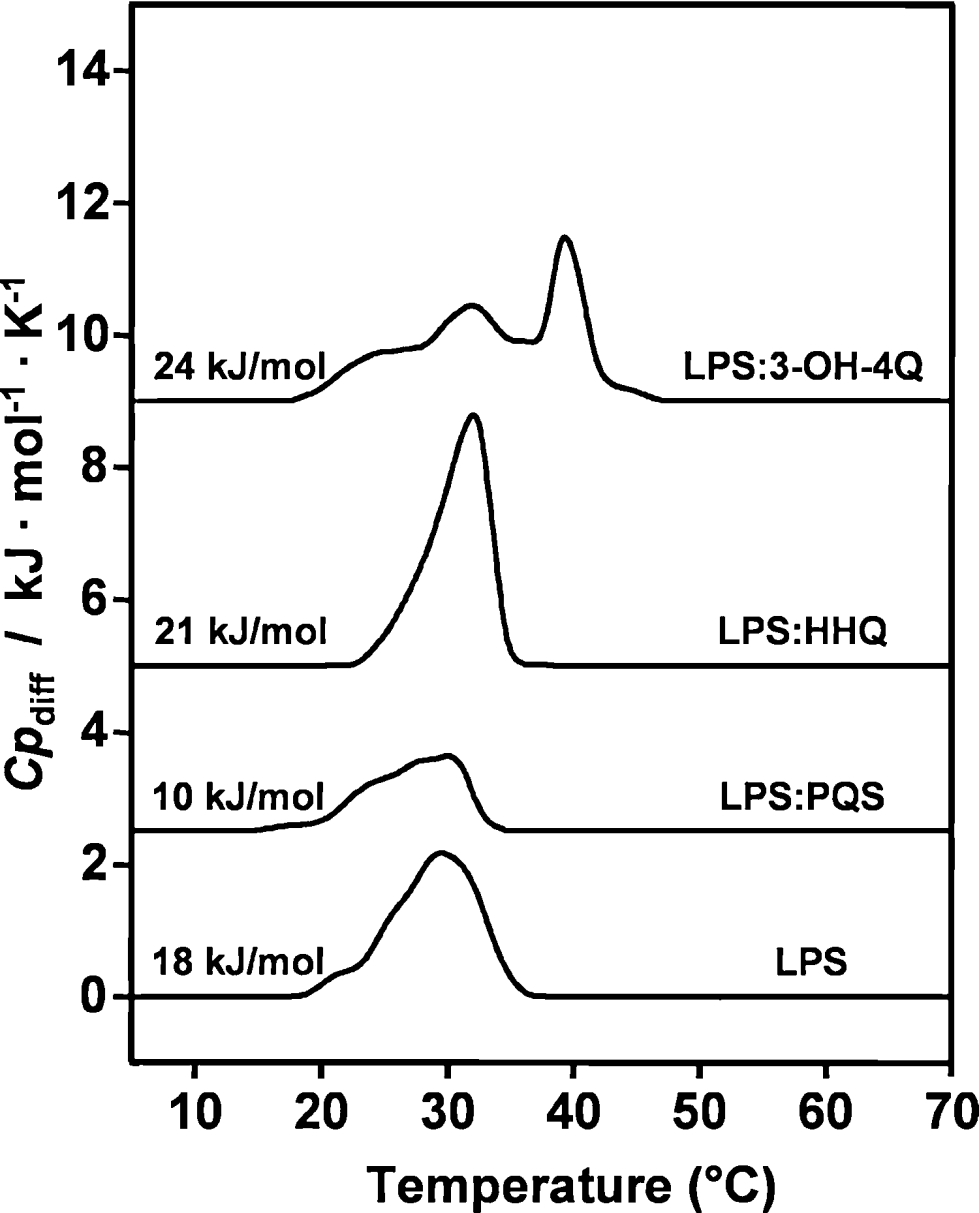
Thermodynamic studies of the phase transition of LPS in the presence of PQS and HHQ. Differential calorimetric scans of *S. minnesota* R60 LPS in the absence and presence of an equimolar amount of PQS, HHQ and 3OH-4Q. Heating scans were performed from 10°C to 80°C with a heating rate of 1°C min^−1^. Plotted is the specific molar excess heat capacity versus temperature. Data represent at least three separate scans.

The transition of LPS from an ordered gel-like state to an unordered liquid crystalline state can also be assessed using differential scanning calorimetry (DSC). DSC examines the amount of energy required for LPS to complete its phase transition. The heat capacity curve of *S. minnesota* R60 LPS is characterized by an endothermic phase transition representing the transition from a gel into the liquid crystalline phase (Fig. 3). The corresponding phase transition enthalpy was 18 kJ mol^−1^, with a heat capacity maximum at 31°C. The presence of PQS reduced the phase transition enthalpy to 10 kJ mol^−1^, and the maximum of the heat capacity was slightly shifted to a lower temperature (30.4°C). This result indicates a strong reduction of the van der Waals interaction between the hydrocarbon chains of the lipids as a result of the presence of PQS. The IR investigation of the thermotropic phase properties of an equimolar ratio of LPS : PQS indicated an additional increase of the gauche isomers between 50°C and 55°C. This transition was, however, not accompanied by a change in the heat capacity curve. In contrast to the effect induced by PQS, the presence of HHQ shifted the maximum of the heat capacity curve to ∼32.5°C, with a slight increase of the phase transition enthalpy to 21 kJ mol^−1^ (Fig. 3). The lower phase transition enthalpy observed upon PQS addition corroborates the IR data demonstrating that PQS decreases the acyl chain fluidity of lipid A.

In order to elucidate the importance of the PQS alkyl chain for biological activity, a PQS derivative that does not contain an alkyl chain (3-hydroxy-4-quinolone or 3OH-4Q, Fig. 1B) was also investigated. The addition of 3OH-4Q considerably broadened the coexistence of the LPS phase transition (Fig. 3), indicating that two maxima in the heat capacity were observed, one at ∼32°C and another at ∼39°C. The former is located close to the maximum of the heat capacity curve of the pure LPS system. The second maximum is indicative of a strong interaction of the lipid head group with the PQS derivative. The overall phase transition enthalpy was increased to 24 kJ mol^−1^. The reproducibility of the phase behaviour for the system LPS : 3OH-4Q was demonstrated by running up to five heating scans, which were in each case identical. Comparing the calorimetric results of PQS with 3OH-4Q indicated that the PQS alkyl chain induced a destabilization and perturbation of the lipid hydrocarbon chain organization, which is associated with a decrease of the phase transition enthalpy.

### PQS alters the hydration and mobility of the 4′-phosphate of *P. aeruginosa* lipid A

**Fig. 4:**
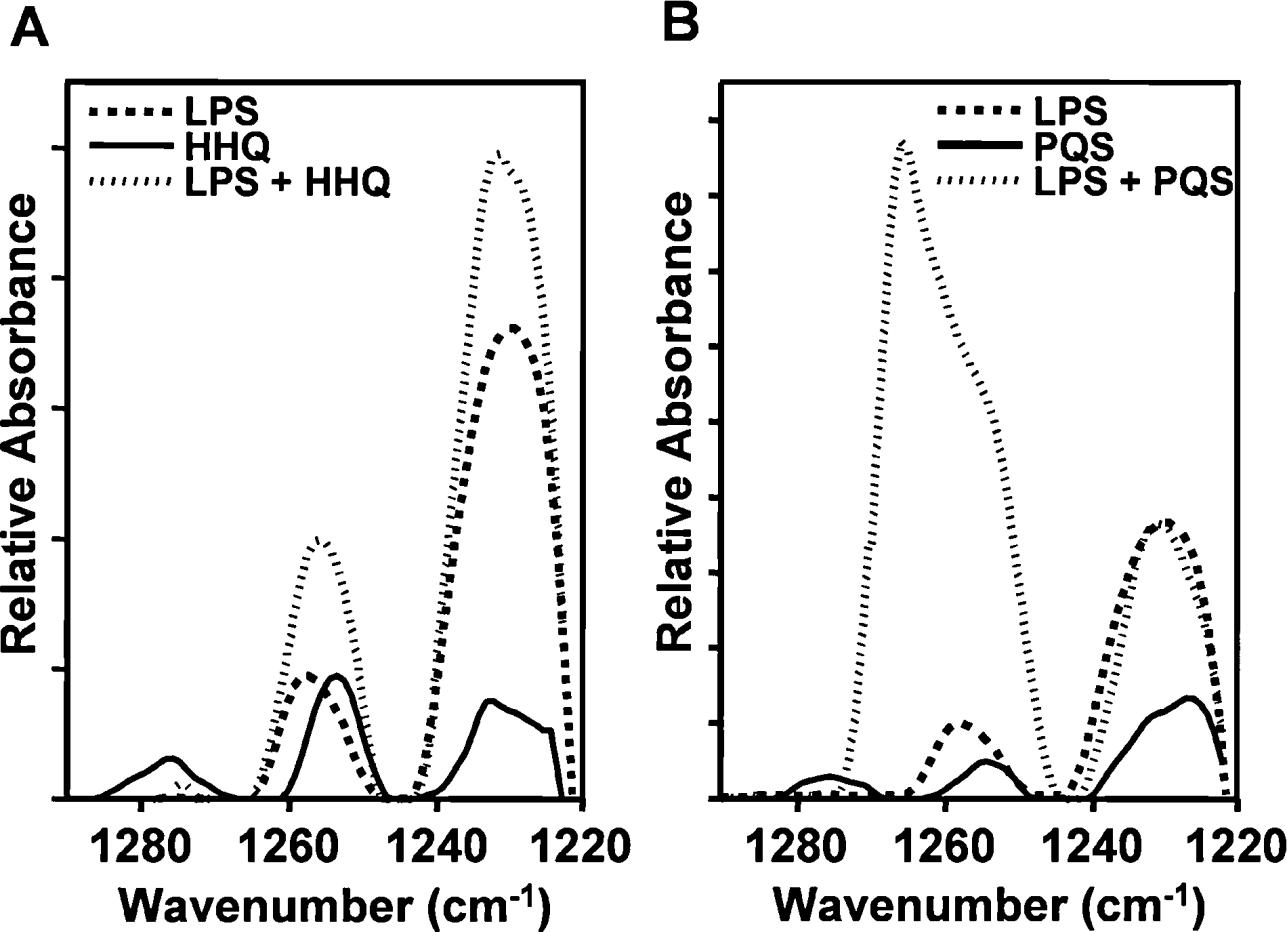
Interaction of PQS with the 4′-phosphate of lipid A. Infrared spectra in the wave number range of 1300–1220 cm^−1^ for *P. aeruginosa* LPS with and without PQS and HHQ. This range corresponds to the anti-symmetric stretch of the phosphate groups of lipid A. The characteristic wave numbers for the 4′- and 1-phosphates are 1260 and 1230 cm^−1^ respectively. LPS alone, quinolones alone or a LPS : quinolone mixture was evaporated on an ATR crystal and the vibrational spectra measured. A. The addition of LPS alone and HHQ alone absorbancies were equivalent to the absorbance of LPS + HHQ indicating no significant reaction between HHQ and LPS. B. The absorbance of PQS + LPS was significantly increased compared with each alone at the 1260 cm^−1^ peak, and the peak was strongly shifted indicating that PQS interacted strongly with the 4′-phosphate of lipid A. The spectra were obtained after base-line subtraction. This datum represents duplicate measurements.

Our analysis with *S. minnesota* R60 LPS indicated a strong interaction between PQS and LPS. Although these results provide new information regarding LPS : PQS interactions, it is critical to examine PQS interactions with native *P. aeruginosa* LPS. The techniques utilized thus far are not feasible with *P. aeruginosa* LPS as a result of its high fluidity as compared with *S. minnesota* R60 (data not shown); thus we utilized a second IR technique (attenuated total reflectance, or ATR) which allowed examination of PQS with specific *P. aeruginosa* LPS constituents. For these experiments, aqueous suspensions of *P. aeruginosa* LPS with and without PQS and HHQ were examined by ATR. The IR data (spectral range 1800–900 cm^−1^) of the pure quinolones is shown in Fig. S1. The spectra of HHQ alone and in the presence of LPS were nearly identical (indicating little interaction), whereas those of PQS alone and in the presence of LPS largely differed (Fig. 4A and B, Fig. S1), indicating a strong interaction.

To study the influence of the quinolones on specific *P. aeruginosa* lipid A functional groups, the spectral range of 1280–1220 cm^−1^ was monitored (Fig. 4A and B). In this range, the bands corresponding to the asymmetric stretch of the negatively charged phosphates 
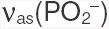
 are found. For LPS, two bands at 1260 and 1230 cm^−1^ are characteristic of the 4′-phosphate and 1-phosphate, respectively, of the lipid A moiety (Brandenburg *et al*., 1997). The LPS lipid A backbone has a strong inclination of the plane of the diglucosamine bisphosphoryl headgroup having an angle of 45–55° with respect to the membrane plane (perpendicular to the direction of the acyl chains) (Seydel *et al*., 2000). Thus, the 4′-phosphate is buried in the hydrophobic moiety between neighbouring LPS molecules, whereas the 1-phosphate projects outwards into the hydrophilic environment. Addition of the spectra corresponding to HHQ and LPS alone are equivalent to that of the LPS : HHQ mixture, thus indicating that HHQ exhibits little interaction with either phosphate (Fig. 4A). In contrast, addition of the PQS and LPS spectra drastically differ from the PQS : LPS complex, particularly in regard to the 4′-phosphate (Fig. 4B). These results indicate that PQS interacts strongly with the 4′-phosphate in the hydrophobic moiety by making it more hydrated and mobile (indicated by a peak shift and increase in peak height respectively), while little interaction is observed with the 1-phosphate. Removal of the PQS alkyl side-chain (3OH-4Q) eliminated this interaction (data not shown). These data along with Figs 2 and 3 demonstrate that PQS, but not HHQ, interacts strongly with LPS from *P. aeruginosa* and *S. minnesota* R60.

### PQS incorporates into LPS and phospholipids

**Fig. 5:**
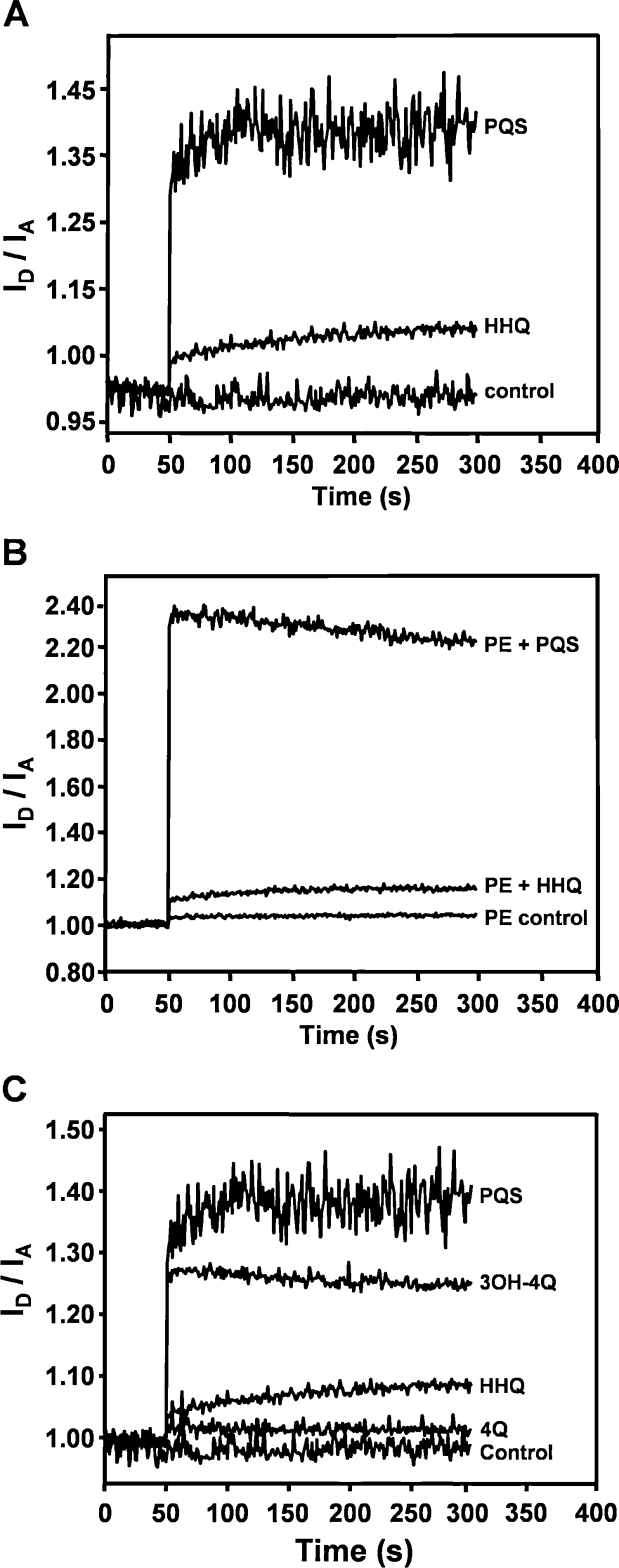
PQS interaction with *P. aeruginosa* LPS and phospholipids. FRET spectroscopic signal I_D_/I_A_ versus time for (A) LPS aggregates, (B) PE liposomes alone and with addition of PQS and HHQ, (C) LPS aggregates with the addition of PQS and HHQ derivatives. Labelled LPS or PE were added at *t* = 0 s, and the quinolones were added after 50 s. The signal was then measured for an additional 250 s. As FRET is used here as a probe dilution assay, intercalation of PQS and HHQ caused an increase of the distance between acceptor and donor, therefore leading to reduced energy transfer. As a control, methanol was added without quinolones. Data represent triplicate measurements. Data similar to PE were obtained with PG (data not shown).

The Gram-negative outer membrane is composed of an outer leaflet of LPS and an inner leaflet of phospholipids. Although our data indicate that PQS interacts with LPS, it is conceivable that PQS may also interact with phospholipids within the outer membrane. To examine this, incorporation of PQS and HHQ into *P. aeruginosa* LPS aggregates or phospholipids was measured by fluorescence resonance energy transfer (FRET). The test was performed as a probe dilution assay in which *P. aeruginosa* LPS and the phospholipids phosphatidylethanolamine (PE) and phosphatidylglycerol (PG) were mixed with the donor-conjugated PE (4-nitro-benz-2-oxa-phosphatidylethanolamine, NBD-PE) and acceptor-conjugated PE (Rhodamine-phosphatidylethanolamine, Rh-PE), and the quinolones were added. As FRET is used here as a probe dilution assay, intercalation of PQS and HHQ causes an increase of the distance between acceptor and donor, therefore leading to reduced energy transfer. For LPS aggregates, there was a strong decrease of the FRET signal upon addition of PQS, while a significantly smaller change was observed for HHQ (Fig. 5A). This suggests that PQS more readily, compared with HHQ, incorporates into and/or fuses with LPS aggregates, thus increasing the distance between the donor and acceptor labels. This provides further evidence for the interaction of LPS with the biologically active quinolone PQS, but not the inactive derivative HHQ. Removal of the PQS alkyl side-chain (3OH-4Q) signal decreased the FRET compared with HHQ, although not to the same level as PQS (Fig. 5C). Similar experiments with phospholipid liposomes made from zwitterionic PE and negatively charged PG indicated that as for LPS, PQS readily incorporates into PE as well as PG liposomes (Fig. 5B). These data indicate that PQS interacts with the primary lipid membrane components of the bacterial outer membrane, and that the third position hydroxyl and alkyl side-chains are important for the interaction with LPS.

### The PQS : LPS interaction is endothermic

**Fig. 6:**
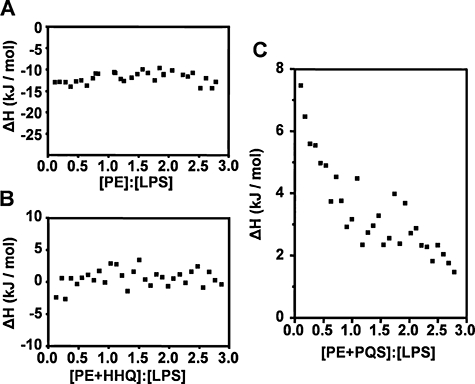
Thermodynamic interactions of LPS and PQS. Isothermal titration of (A) pure PE, (B) PE + HHQ and (C) PE + PQS mixtures into *P. aeruginosa* LPS aggregates. Quinolones were added to PE to increase solubility. Three microlitres of PE alone or PE : quinolone mixtures were titrated into the LPS-containing cell every 5 min. Measurements were performed at 37°C with constant stirring. Values plotted for HHQ and PQS experiments (B and C) represent values after subtraction of PE alone measurements (A). Data represent triplicate measurements.

Our data thus far reveal specific LPS : PQS interactions that result in ‘ordering’ of LPS, although the thermodynamic properties of this interaction are not known. To examine the thermodynamic properties of quinolones with LPS, isothermal titration calorimetry (ITC) was utilized. For these experiments, quinolones were incorporated into a lipid matrix (i.e. PE liposomes) as a result of their insolubility in aqueous media. The titration of PE into LPS led to a significant exothermic reaction (negative enthalpy changes), with little fluctuation in the applied concentration range (Fig. 6A). The addition of the PE : HHQ mixture caused a low enthalpic reaction, again with very little fluctuation (Fig. 6B). In contrast, the PE : PQS mixture caused a strong endothermic reaction, which decreased considerably in the observed concentration range as a result of the saturation of the PQS : PE interaction with LPS (Fig. 6C). The endothermic nature (decrease in entropy) of this reaction correlates with the IR and DSC data demonstrating a decrease in lipid A acyl chain fluidity upon PQS addition. Not only does this datum suggest that the PQS : LPS interaction is endothermic, but also that PQS possesses higher affinity for LPS than phospholipids as it exhibits significant interaction with LPS in the presence of phospholipids.

### PQS : LPS aggregates display unique structure

**Fig. 7:**
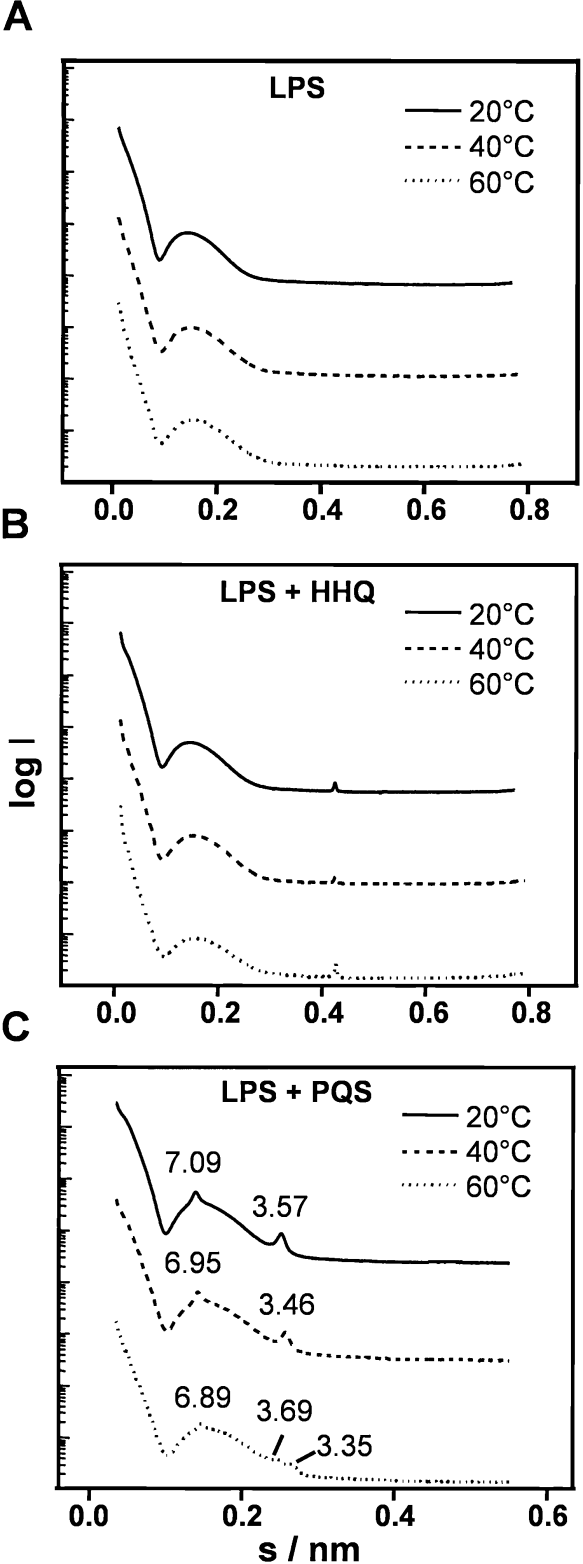
Multilamellar stacking of LPS in the presence of PQS. SAXS patterns of *S. minnesota* R60 LPS (A) alone, (B) in the presence of HHQ (5:1 ratio LPS : HHQ), or (C) in the presence of PQS (5:1 ratio LPS : PQS). Plotted is the logarithm of the scattering intensity versus scattering vector *s* = 1/*d*(2 sin *θ/λ*). (*d* = spacing, *θ* = scattering angle, wave length (*λ*) = 0.15 nm). Data represent duplicate measurements.

Although it is clear that PQS interacts with LPS, the aggregate structure of PQS : LPS is unknown. To examine the influence of PQS and HHQ on the aggregate structure of *S. minnesota* LPS, small-angle X-ray scattering (SAXS) using synchrotron radiation was used to examine the diffraction patterns of pure LPS and LPS in the presence of HHQ and PQS. Regarding Braggs law, a mutlilamellar stacking with a repeat distance (d) results in a scattering pattern that consists of maxima in the intensities for the scattering vector (s) at the positions of 1/d (first order, periodicity), 2/d (second order) and the like. For these studies, the first two peaks were examined. LPS alone showed a broad diffraction band between 0.1 and 0.35 s nm^−1^, which corresponded to the form factor of a lipid bilayer (Fig. 7A). As can be seen for the LPS : HHQ mixture, this pattern did not change, thereby ruling out a change of the LPS aggregate structure by HHQ (Fig. 7B). In the presence of PQS, however, sharp reflections were observed, e.g. at 40°C at 6.95 nm, resulting from a lamellar stacking with the first order (periodicity) and 3.46 nm with the second order reflection (Fig. 7C). Regarding the values of the periodicity, it should be emphasized that the action of PQS led to a dramatic reduction of the periodicity (6.95 nm) as compared with pure LPS, for which values around 10 nm have been found (Seydel *et al*., 1993b). Similar results were obtained when the interaction of the quinolones with the isolated *S. minnesota* lipid A was examined. A change of the diffraction patterns leading to sharp reflections characteristic for a multilamellar stacking was also observed only for PQS (data not shown).

### PQS induces liposome-like LPS structures

**Fig. 8:**
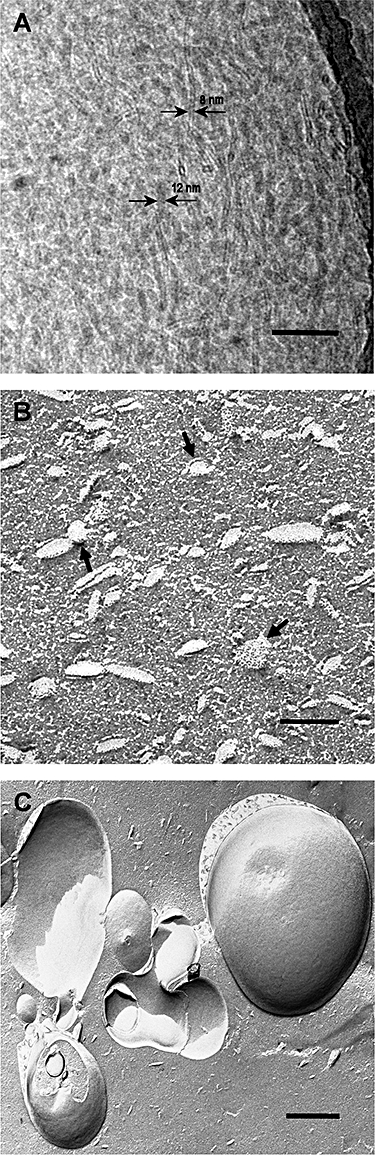
Cryo transmission and freeze-fracture electron micrographs of LPS from *S. minnesota* R60 in the absence and presence of HHQ and PQS. Samples were prepared as described in *Experimental procedures*. A. LPS from *S. minnesota* R60 showing the 8–12 nm bilayer thickness (arrows) (scale bar = 100 nm). B. When PQS was added to LPS, small liposome-like structures with diameters of 20–70 nm were formed (arrows) (scale bar = 100 nm). C. In contrast, the LPS : HHQ mixture formed large micelle structures (100 nm−1 μm) and bilayer fragments (scale bar = 500 nm).

To visually examine the impact of PQS on LPS structure, we performed cryo-transmission electron microscopy (cryo-TEM). In pure form, *S. minnesota* LPS R60 adopts fibrillary structures of different lengths (Fig. 8A). These fibrilles are obviously not completely circular in cross-section showing a ribbon-like appearance with a smaller width of about 8–9 nm and a larger width of about 11–12 nm. The smaller dimension of 8–9 nm corresponds to the bilayer thickness of LPS R60 and is in agreement with former data of SAXS diffraction (Seydel *et al*., 1993b). The addition of PQS caused clumping of LPS into predominantly small clod- or disc-like structures. In many cases, these structures resembled MVs in regards to size (∼50 nm) and shape (Fig. 8B). Rarely bilayer fragments larger than 100 nm in size were observed. For LPS in the presence of HHQ, there was a mixture of several structures, including large vesicles (ranging from 100 nm to larger than 1 μm), small disc-like micelles and bilayer fragments (Fig. 8C), but also large three-dimensional layered aggregates (not shown here). The addition of the PQS derivative 3OH-4Q caused LPS aggregates to form loosely arranged perforated lamellae (Fig. S2). In most instances, linear lamellar fragments are present and not the circular structures formed by PQS.

## Discussion

This study provides a comprehensive examination of the interaction of *P. aeruginosa* quinolones with Gram-negative outer membrane lipids. From these studies, it is clear that the signalling molecule PQS interacts strongly with the lipid A component of LPS from *S. minnesota* R60 and *P. aeruginosa*. The interaction with both LPS molecules is not surprising given the structural similarities between *S. minnesota* R60 and *P. aeruginosa* lipid A (Fig. 1A), and the observation that PQS stimulates MV formation in other Gram-negative bacteria (data not shown), including *S. minnesota* R60 (Fig. S3). The observation that HHQ, which differs from PQS by the absence of the third position hydroxyl (Fig. 1B), exhibits significantly less interaction with LPS, suggesting a critical role of this hydroxyl in mediating interactions with lipid A. These results also provide insight into why HHQ does not stimulate MV formation in *P. aeruginosa* and demonstrates the structural specificity of *P. aeruginosa* quinolones. This latter point is intriguing as *P. aeruginosa* produces over 50 quinolone molecules with structural similarity to PQS (Deziel *et al*., 2004); however, only PQS naturally stimulates MV formation (Mashburn and Whiteley, 2005).

The IR data presented are indicative of a dramatic interaction of LPS with PQS, leading to a change of vibrational bands of the quinolone as well as of LPS. In particular, the phase transition changes led to a significant broadening concomitant with a considerable decrease of phase transition enthalpy (Fig. 2). PQS readily incorporated into LPS aggregates as well as into phospholipid liposomes, which is a proof that the interaction is governed by hydrophobic forces (Fig. 5). However, there is also a non-hydrophobic interaction as shown by the change of the 4′-phosphate vibrational band upon PQS addition (Fig. 4). This additional interaction with LPS enhances the affinity of PQS for LPS as compared with phospholipids.

An intriguing aspect of *P. aeruginosa* quorum signalling is the utilization of MVs as trafficking vehicles for PQS. As previously hypothesized (Mashburn-Warren and Whiteley, 2006), our results provide strong evidence that PQS readily associates with LPS and is likely embedded within the LPS component of MVs as opposed to the phospholipid component or the aqueous interior. The freeze-fracture micrographs provide insight into why PQS-, but not HHQ-induced MVs are competent for signal trafficking. Our results indicate that HHQ interacts with LPS, although not to the same degree as PQS. This observation can be seen with the freeze-fracture data where HHQ formed large micelle-like structures. This was an interesting observation as the SAXS data showed no interaction between LPS and HHQ. It seems that HHQ may interact with LPS; however, this interaction is very different from PQS and ultimately does not lead to MV formation. It is easily intelligible from these results that the extremely large micelle structures of the LPS : HHQ mixture are not suitable for transport (Fig. 8C), regarding the fact that they are larger than whole bacteria. It seems that rather, the small liposome-like structures observed for LPS : PQS would be much more suitable for a target-oriented transport (Fig. 8B). Why HHQ induces larger LPS micelles is unknown, but implicates the third position hydroxyl of PQS as an important component for inducing the curvature necessary for MV-sized LPS aggregates. In addition, LPS formed linear-shaped structures in the presence of 3OH-4Q instead of the circular structures observed in the presence of PQS (Fig. S2), thus also implicating the PQS alkyl side-chain as important for producing MV-like LPS aggregates. Of course, one caveat to this experimental design is that the structures formed in these experiments are composed entirely of LPS, whereas naturally produced MVs are bilayered with an inner leaflet of phospholipid and an outer LPS leaflet. Regardless, these results indicate that through interaction with LPS, PQS induces the curvature necessary to form LPS liposomes with dimensions similar to natural MVs. These data are in accordance with the SAXS data indicating some lamellar stacking of LPS, thus producing a liposome-like structure (Fig. 7).

Examination of LPS interactions with HHQ and and PQS derivatives demonstrate that the alkyl chain and third position hydroxyl mediate PQS interactions with LPS. Specifically, the alkyl chain interacts with the lipid A acyl chains while the third position hydroxyl mediates interactions with the 4′-phosphate. The PQS alkyl chain/LPS acyl chain interactions are most assuredly hydrophobic interactions, as definitive changes in van der Waals interactions between lipid hydrocarbons are observed upon PQS addition (Fig. 3). FRET experiments also provide evidence that the PQS alkyl chain is critical for interaction with LPS, as 3OH-4Q showed reduced integration into LPS (Fig. 5C). These data correlate well with the ability to stimulate MV production, as 3OH-4Q requires twice the concentration of PQS to stimulate MV production in *P. aeruginosa* (Fig. S4). Interaction between the third position hydroxyl of PQS and the 4′-phosphate are not as clear. As previously discussed, the 4′-phosphate is buried in the hydrophobic moiety between neighbouring LPS molecules, and the ATR data clearly show that it becomes more hydrated and mobile upon interaction with PQS (Fig. 4). This likely indicates movement of the 4′-phosphate away from the hydrophobic pocket. The exact nature of the interaction that mediates this increased hydration and mobility is unknown, but a possibility is hydrogen bonding between the third position hydroxyl of PQS and the 4′-phosphate. This possibility is supported by the observation that no interaction was observed between HHQ (lacking the third position hydroxyl) and the 4′-phosphate (Fig. 4A). PQS could also be affecting the 4′-phosphate through interaction with divalent cations (Mg^2+^ and Ca^2+^) in the LPS leaflet of the outer membrane. Divalent cations stabilize the Gram-negative outer membrane by forming salt bridges between negatively charged phosphates of neighbouring LPS molecules. Based on recent observations that PQS has been shown to ‘entrap’ iron (Bredenbruch *et al*., 2006; Diggle *et al*., 2007), it is plausible that PQS could sequester Mg^2+^ and Ca^2+^, thereby affecting the mobility of the 4′-phosphate of lipid A.

As with many bacteria, *P. aeruginosa* modifies its LPS based on environmental conditions. Indeed, *P. aeruginosa* clinical isolates from the lungs of cystic fibrosis patients add 4-amino-4-deoxy-L-arabinose to the 1- or 4′-phosphates (or both) of lipid A (Ernst *et al*., 1999). How these modifications impact PQS interaction with LPS and MV production by *P. aeruginosa* is unknown, but results from this study would suggest that modification of the 4′-phosphate may alter these processes. What is clear from these studies is that both the alkyl chain and third position hydroxyl of PQS are critical for LPS interaction and MV formation.

The observation that addition of PQS causes LPS to retain a more ordered (gel-like) state provides clues to the mechanism of MV formation. The outer membrane of Gram-negative bacteria is stabilized by cross-links to the underlying peptidoglycan; however, these cross-links are not uniform, permitting regions of the outer membrane to bulge away from the bacterium. If the appropriate curvature is reached, this bulging leads to MV formation. *P. aeruginosa* LPS is highly anionic and extremely fluid compared with other Gram-negative bacteria (Rocchetta *et al*., 1999; Rodriguez-Boulan *et al*., 2005), and we hypothesize that this high fluidity likely prevents development of the curvature necessary for MV formation. PQS solves this conundrum by decreasing the fluidity of *P. aeruginosa* LPS through production of PQS and its interactions with lipid A. *P. aeruginosa* is therefore unique in that it requires PQS for production of high levels of MVs. It is intriguing that PQS, a quorum-signalling molecule important for global gene regulation, also mediates an important biological process, such as MV formation through a signal-independent mechanism. Whether or not this self-packaging within the outer membrane is specific to PQS or is critical for other hydrophobic bacterial QS signals is unknown, but these studies provide novel mechanistic insights into an important and understudied biological phenomenon.

## Experimental procedures

### Lipids and quinolones

Lipopolysaccharides from the rough mutant *S. minnesota* strain R60 and *P. aeruginosa* strain PA14 were extracted by the phenol/chloroform/petrol ether method (Galanos *et al*., 1969), from bacteria grown at 37°C, purified and lyophilized. The phospholipids PE from *Escherichia coli* and PG from phosphatidylcholine were purchased from Sigma. All lipid samples were prepared as aqueous suspensions in 20 mM Hepes, pH 7.4. The lipids were suspended directly in buffer and were temperature-cycled three times between 5°C and 70°C and then stored for at least 12 h before measurement. To guarantee physiological conditions, the water content of the samples was usually around 95%.

For preparations of liposomes, PE and PG were solubilized in chloroform, and the solvent was evaporated under a stream of nitrogen. The lipids were re-suspended in the appropriate volume of Hepes buffer, and treated as described above (temperature cycling). The resulting liposomes were large and multilamellar as detected in some electron microscopic experiments (kindly performed by H. Kühl, Division of Pathology, Forschungszentrum Borstel). All quinolones were purchased from Synthech Solutions (San Diego, CA). Quinolones were either re-suspended in a 1:1 mix of ethyl acetate : acetonitrile or methanol.

### Isolation and quantification of MVs

A *pqsA/pqsH* double mutant in *P. aeruginosa* PA14 was constructed by the insertion of aacC1 into the pqsH coding region (Mashburn and Whiteley, 2005) of the *pqsA* Tn5 mutant (Mashburn *et al*., 2005). For MV preparation, an overnight culture of *P. aeruginosa* pqsA^-^H^-^ was diluted to an OD_600_ of 0.05 in MOPS minimal media (Mashburn *et al*., 2005) containing 10 mM tyrosine and 10 mM phenylalanine and allowed to grow at 37°C with shaking at 250 r.p.m. for 15 h. Quinolones were then added for 5 h and MV formation quantified. *S. minnesota R60* was diluted to an OD_600_ of 0.05 in MOPS minimal media containing 20 mM glucose with and without the addition of 50 μM PQS or HHQ and allowed to grow at 37°C with shaking at 250 r.p.m. for 7 h. MVs were purified as described previously (Kadurugamuwa and Beveridge, 1995), except that ultracentrifugation was performed at 265 000 *g* for 60 min in a Beckman 70.1 Ti rotor. For quantitative purposes, MV preparations were subjected to one buffer exchange with Nanosep 300 kDa omega centrifugal devices (Pall Life Sciences). Protein content of the MVs was used as a measure of MV levels using the Coomassie Plus Protein assay (Pierce Biotechnology, Rockford, IL).

### Fourier-transform infrared spectroscopy

The infrared spectroscopic measurements were performed on an IFS-55 spectrometer (Bruker, Karlsruhe, Germany). The lipid samples were placed in a CaFB_2B_ cuvette with a 12.5 μm Teflon spacer. Temperature scans were performed automatically between 10°C and 70°C with a heating rate of 0.6°C min^−1^. Every 3°C, 200 interferograms were accumulated, apodized, Fourier transformed and converted to absorbance spectra. For strong absorption bands, the band parameters (peak position, band width, band intensity) were evaluated from the original spectra, if necessary after subtraction of the strong water bands. The main vibrational band used for the analysis were the asymmetrical stretching vibration of the methylene groups ν_sB_(CH2) located around 2850 cm^−1^, a measure of order of the lipid A chains, and the anti-symmetric stretching vibrations bands of the negatively charged phosphate groups 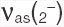 in the range 1280–1200 cm^−1^ (Brandenburg *et al*., 1997).

### Differential scanning calorimetry

Differential scanning calorimetry measurements were performed with a MicroCal VP scanning calorimeter (MicroCal, Northhampton, MA, USA). The heating and cooling rates were 1°C min^−1^. Heating and cooling curves were measured in the temperature interval from 10°C to 80°C. The phase transition enthalpy was obtained by integration of the heat capacity curve as described previously (Jurgens *et al*., 2002). Usually, three consecutive heating and cooling scans were measured. The lipids as well as the quinolones were dissolved in an organic solvent composed of acetyl nitrile and ethyl acetate 1:1 v/v. Lipid to quinolone at a 1:1 molar ratio was prepared by pipetting the corresponding amount of quinolone to the lipid. The organic solvent was rapidly removed by gently heating the sample to 30°C under a nitrogen stream. After solvent evaporation, the samples were kept over night under vacuum to remove solvent traces. The DSC samples were prepared by dispersing the sample in PBS buffer at pH 7.4. The lipid dispersion was prepared according to recently described protocols at a concentration of approximately 2 mg ml^−1^ (corresponds to 0.5 mM).

### isothermal titration calorimetry

Microcalorimetric measurements of the binding of the quinolones to LPS were performed on a MCS isothermal titration calorimeter (Microcal, Northampton, MA, USA) at 37°C. *P. aeruginosa* LPS samples (0.1 mM) were dispensed into the microcalorimetric cell (volume 1.5 ml). As the quinolones are not soluble in aqueous buffers, they were co-solubilized with PE. This mixture was filled into the syringe compartment (volume 100 μl), each after thorough degassing of the suspensions by ultrasonification. After temperature equilibration, the PE : quinolone mixtures were titrated in 3 μl of portions every 5 min into the LPS-containing cell under constant stirring, and the measured heat of interaction after each injection measured by the ITC instrument was plotted versus time. The titration curves were repeated three times. As a control, pure PE was titrated into the LPS dispersion.

### X-ray diffraction

X-ray diffraction measurements were performed at the European Molecular Biology Laboratory outstation at the Hamburg synchrotron radiation facility hasylab using the SAXS camera X33 (Koch, 1988). Diffraction patterns in the range of the scattering vector 0.1 < *s* < 1.0 nmP^−1P^ (*s* = 2 sin *θ/λ*, *2θ* scattering angle and *λ* the wave length = 0.15 nm) were recorded at 40°C with exposure times of 1 min using an image plate detector with online readout (MAR345, MarResearch, Norderstedt/Germany) (Roessle *et al*., 2007). The *s*-axis was calibrated with Ag-behenate which has a periodicity of 58.4 nm. The diffraction patterns were evaluated as described previously (Brandenburg, 1993) assigning the spacing ratios of the main scattering maxima to defined three-dimensional structures. The lamellar and cubic structures are most relevant here. They are characterized by the following features:

- lamellar: the reflections are grouped in equidistant ratios, i.e. 1, 1/2, 1/3, 1/4 etc. of the lamellar repeat distance dB_lB_;
- cubic: the different space groups of these non-lamellar three-dimensional structures differ in the ratio of their spacings. The relation between reciprocal spacing *s*_*hklB*_ = 1/d_hklB_ and lattice constant *a* is

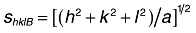


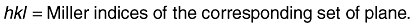

### Freeze fracture and cryo-TEM

*Salmonella minnesota* R60 LPS and LPS : quinolone mixtures were prepared as described above at 90% water content (3–5 mg per 30 μl). For cryo transmission experiments, 3 μl of the dispersion was placed on a copper grid with perforated carbon film (Quantifoil R 1.2/1.3, Jena, Germany), and excess liquid was blotted automatically for 2 s between two strips of filter paper. Subsequently, the samples were rapidly plunged into liquid ethane (cooled to ∼−175°C) in a cryo-box (Zeiss, Oberkochen). Excess ethane was removed with a piece of filter paper. The sample was transferred with a liquid nitrogen-cooled holder (Gatan 626, München, Germany) into the cryo-TEM (Philips CM 120, Netherlands) and investigated at 120 kV. The micrographs were generated by a Tietz-Fast Scan CCD Camera (TVIPS, Gauting, Germany).

For freeze fracturing, the samples, copper sandwich profiles and instruments for manipulation, were incubated at room temperature or at 40°C. A small amount of the sample was sandwiched between two copper profiles as used for the double-replica technique and frozen by plunging the sandwiches immediately into liquefied ethane/propane mixture cooled in liquid nitrogen. Fracturing and replication were performed at −150°C in a BAF 400T freeze-fracture device (BAL-TEC, Liechtenstein) equipped with electron guns and a film sheet thickness monitor. The replicas were placed on copper grids, cleaned with a chloroform–methanol mixture, and examined in an EM 901 electron microscope (Zeiss, Oberkochen, Germany).

### Fluorescence resonance energy transfer spectroscopy

Intercalation of the quinolones into *P. aeruginosa* LPS aggregrates or into liposomes made from PE or PG was determined by FRET spectroscopy applied as a probe dilution assay (Schromm *et al*., 1996). To the aggregates or liposomes, which were labelled with the donor dye NBD-PE and acceptor dye Rh-PE, the quinolones were added, all at final molar ration of 1:10, LPS or PE : quinolones. Intercalation was monitored as the increase of the ratio of the donor intensity I_D_ at 531 nm to that of the acceptor intensity I_A_ at 593 nm (FRET signal) over 300 s.

## Supplementary material

This material is available as part of the online article from: http://www.blackwell-synergy.com/doi/abs/10.1111/

j.1365-2958.2008.06302.x

(This link will take you to the article abstract).
