# Supplementary material for: Interaction of quorum signals with outer membrane lipids: insights into prokaryotic membrane vesicle formation
Source: Mol Microbiol. 2008 Jun 9;69(2):491–502. doi: 10.1111/j.1365-2958.2008.06302.x (PMC2615190; doi:10.1111/j.1365-2958.2008.06302.x)
Supplement: Supplementary file 1 [file mmi0069-0491-SD1.pdf]

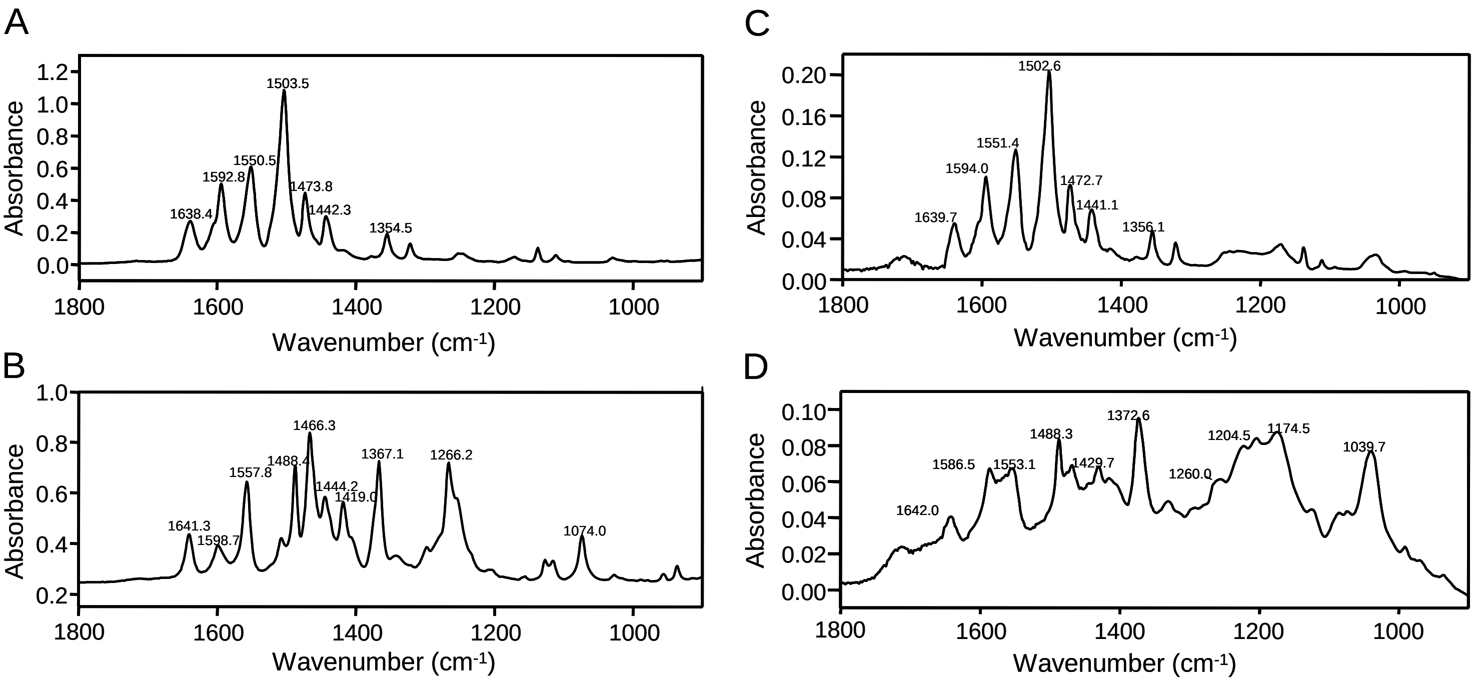

Supplementary Fig. 1. Infrared spectra in the wavenumber range 1800 to 900 cm^{-1} for the pure quinolones PQS and HHQ. Solutions of HHQ (A) and PQS (B) LPS + HHQ (C) and LPS + PQS (D) were evaporated on the ATR crystal and the vibrational spectra measured. Within this range specific functional groups within LPS can be studied. By comparing the quinolones alone and the quinolone:LPS mixture, the influences of the quinolones on LPS and LPS on the quinolones can be examined. HHQ alone and in the presence of LPS were nearly identical, whereas PQS alone and PQS with LPS are drastically different. Data represent duplicate measurements.

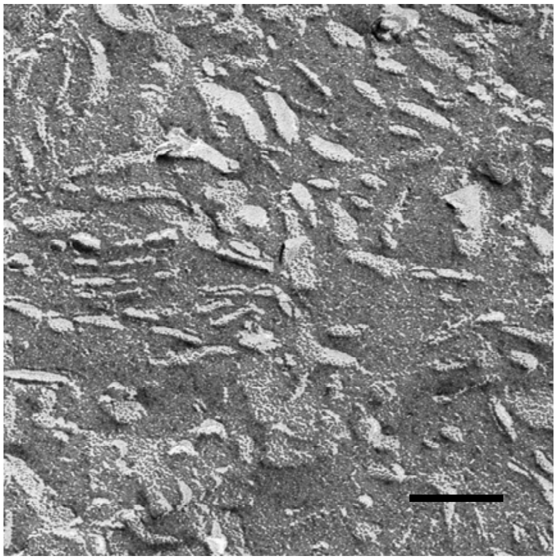

Supplementary Fig. 2. Freeze-fracture electron micrograph of LPS from *S. minnesota* R60 in the presence of 3OH-4Q. Samples were prepared as described in Materials and Methods. Scale bar = 100nm.

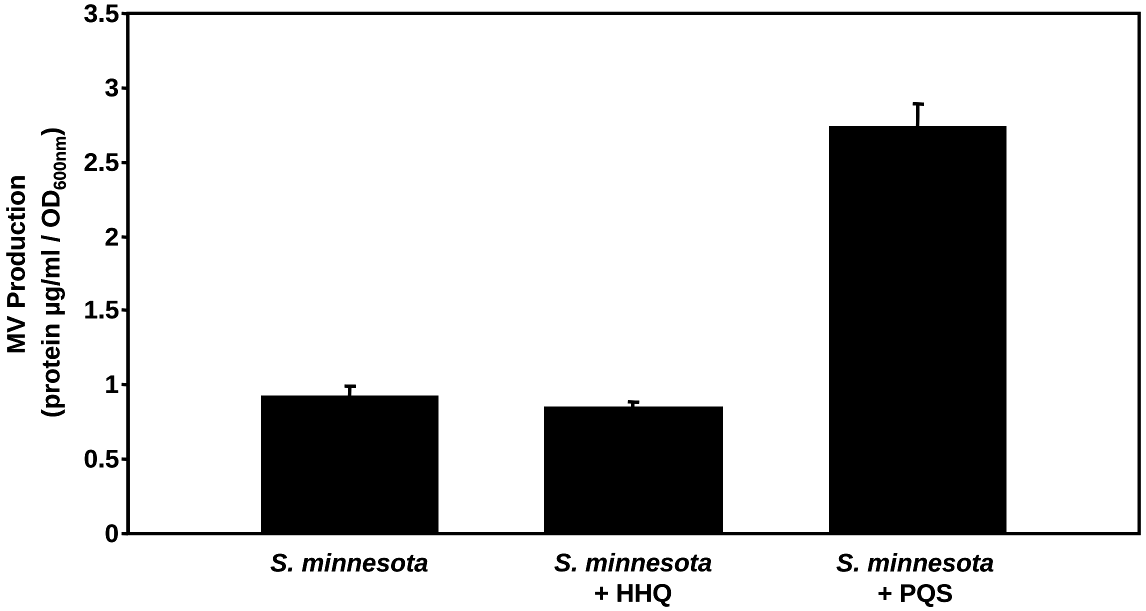

Supplementary Fig. 3. PQS, but not HHQ, stimulates MV formation in *S. minnesota*. *S. minnesota* R60 was grown with and without the exogenous addition of 50 µM PQS or 50 µM HHQ. MV formation was quantified as described in Material and Methods. Error bars represent the standard deviation of duplicate experiments.

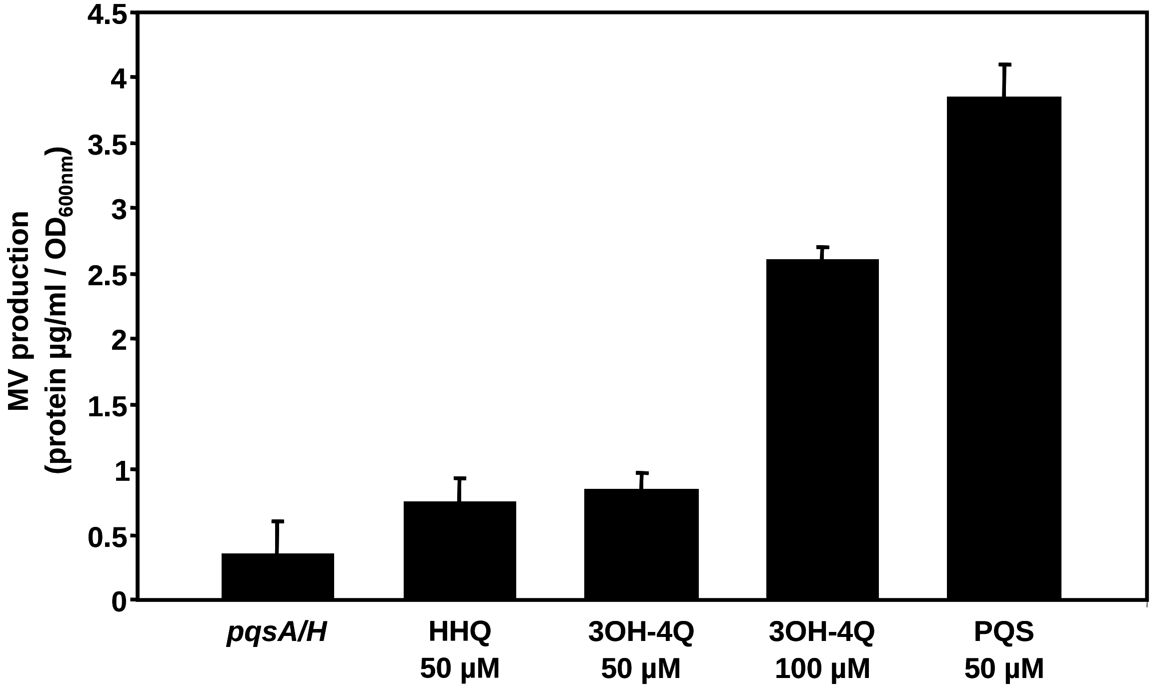

Supplementary Fig. 4. The PQS alkyl chain is important for MV formation. A *P. aeruginosa pqsA⁻H⁻* mutant was grown in MOPS defined media containing 10 mM phenylalanine and 10 mM tyrosine. 50 µM HHQ, 50 µM 3OH-4Q, 100 µM 3OH-4Q, or 50 µM PQS was added and vesicle formation quantified as described in Materials and Methods. Error bars represent the standard deviation for triplicate experiments.
